# Associations of physical fitness and choice reaction time with chronic fatigue in working-age adults: a population-based study

**DOI:** 10.3389/fpubh.2026.1779881

**Published:** 2026-03-17

**Authors:** Xinyi Ma, Mingrui Shao, Chengwei Xu, Weiping Du

**Affiliations:** 1 School of Physical Education, Shanghai Normal University, Shanghai, China; 2 School of Physical Education, Xi'an Fanyi University, Xi An, China; 3 School of Physical Education, Ningxia Normal University, Guyuan, China

**Keywords:** chronic fatigue, logistic regression, occupational group, physical fitness, reaction time, ROC

## Abstract

**Background:**

Chronic fatigue is prevalent among working-age adults and has been linked to reduced physical capacity and impaired cognitive performance. While physical fitness is considered protective against fatigue-related dysfunction, few large-scale studies have examined how multidimensional fitness profiles relate simultaneously to chronic fatigue status and functional cognitive–motor outcomes. Clarifying these associations may improve risk stratification and functional assessment in occupational health settings.

**Methods:**

In this cross-sectional study, 3,025 adults from the National Physical Fitness Health Test were evaluated. Chronic fatigue was defined using a standardized questionnaire cutoff. Physical fitness was assessed using an eight-component standardized battery and summarized as a composite Physical Fitness Index. Choice reaction time (CRT) was measured as a functional cognitive outcome. Multivariable logistic and linear regression models examined independent associations, adjusting for demographic, occupational, and anthropometric covariates. Sex-stratified ROC analyses evaluated discriminatory performance.

**Results:**

Chronic fatigue was identified in 5.45% of participants and varied significantly across occupational groups. Individuals with chronic fatigue demonstrated slower CRT and lower overall physical fitness (both *p* < 0.001). In fully adjusted models (AUC = 0.725), higher physical fitness was strongly associated with lower odds of chronic fatigue. Chronic fatigue remained independently associated with slower CRT after adjustment, whereas higher fitness predicted faster reaction time (β = −11.66 ms per SD; *p* < 0.001). CRT showed moderate discriminatory ability for chronic fatigue in both males (AUC = 0.761) and females (AUC = 0.727).

**Conclusions:**

Chronic fatigue is associated with clinically meaningful slowing of cognitive–motor response and reduced multidimensional fitness. Overall fitness independently relates to both fatigue risk and reaction performance, underscoring the importance of maintaining physical fitness to mitigate functional impairment in working-age populations.

## 1 Introduction

Chronic fatigue represents a significant and persistent health concern characterized by prolonged exhaustion, reduced functional capacity, and impaired quality of life. While severe and long-lasting fatigue may meet clinical diagnostic criteria for myalgic encephalomyelitis/chronic fatigue syndrome (ME/CFS), a much larger proportion of individuals in the general population experience chronic fatigue symptoms that do not necessarily reach formal diagnostic thresholds but nonetheless impose substantial personal, occupational, and societal burdens (1–4). Population-based studies have demonstrated that chronic fatigue symptoms are common among working-age adults and are associated with reduced productivity, increased healthcare utilization, and considerable economic costs.

Extensive clinical and biomedical research has established ME/CFS as a complex, multisystem condition involving immune dysregulation, neuroendocrine disturbances, mitochondrial dysfunction, and altered central nervous system function. Neuroimaging and neurophysiological studies consistently report abnormalities in brain regions involved in cognitive control, sensory integration, and motor planning, providing biological correlates for commonly reported symptoms such as cognitive slowing and brain fog (5, 6). Importantly, these biological alterations are not restricted to clinically diagnosed cases but may also be present along a continuum of fatigue severity in population-based samples (7), underscoring the relevance of investigating chronic fatigue beyond strict clinical case definitions.

From a functional standpoint, chronic fatigue is frequently accompanied by impairments in physical capacity and exercise tolerance. Studies in patients with ME/CFS have documented reduced muscle strength, impaired postural control, and an inability to reproduce peak oxygen uptake during repeated exercise testing, suggesting both peripheral muscular dysfunction and altered central motor drive (8, 9). These functional limitations are thought to arise from a combination of impaired energy metabolism, abnormal fatigue perception, and dysregulated neuromuscular control (6, 10). However, much of the existing evidence is derived from small clinical samples, limiting generalizability to broader working-age populations experiencing chronic fatigue symptoms.

Health-related physical fitness provides an objective framework for assessing physiological reserve across multiple domains, including muscular strength, neuromuscular coordination, flexibility, and cardiorespiratory function (11). Declines in physical fitness have been shown to predict adverse health outcomes, including increased morbidity, mortality, and functional disability, independent of traditional risk factors (12–15). In particular, grip strength has emerged as a robust indicator of overall health status and survival across diverse populations (14). Despite this, the relationship between multidimensional physical fitness and chronic fatigue in large, population-based samples remains insufficiently characterized, with prior studies often focusing on isolated fitness measures rather than integrated functional profiles.

In parallel with physical impairments, cognitive and motor processing deficits are increasingly recognized as central features of chronic fatigue. Slowed information processing, impaired attention, and delayed motor responses are commonly reported in individuals with fatigue-related complaints (5, 6). Choice reaction time, which integrates sensory processing, cognitive decision-making, and motor execution, offers a simple yet sensitive measure of central processing efficiency. Although reaction time slowing has been documented in clinical ME/CFS cohorts, its association with physical fitness and fatigue status in community-based populations has received limited empirical attention.

Moreover, chronic fatigue is shaped by demographic, occupational, and lifestyle contexts. Epidemiological studies indicate that fatigue prevalence varies across sex, age, and occupational groups, with psychosocial stress, sedentary work patterns, and sustained cognitive demands contributing to fatigue vulnerability (7, 16). These contextual factors may interact with individual physiological reserve, such that fatigue emerges when environmental demands exceed available physical or cognitive capacity, a concept consistent with reserve capacity and stress, adaptation models (7). Nevertheless, few studies have examined physical fitness, functional cognitive performance, and occupational characteristics simultaneously within a unified analytical framework.

Therefore, the present study aimed to investigate the associations between multidimensional physical fitness, choice reaction time, and chronic fatigue status in a large sample of working-age adults from Western China. Specifically, we sought to (1) compare physical fitness and reaction performance between individuals with and without chronic fatigue; (2) identify independent physical fitness correlates of chronic fatigue after adjustment for demographic, occupational, and anthropometric factors; and (3) evaluate the discriminatory performance of selected physical fitness and reaction time indicators. By integrating objective physical and cognitive—motor measures, this study seeks to provide a comprehensive functional characterization of chronic fatigue in a population-based setting, bridging clinical insights from ME/CFS research with public health-oriented fatigue assessment.

## 2 Method

### 2.1 Participants

Between September 2022 and November 2024, a total of 3,508 adults (1,638 male and 1,870 female) were randomly selected from a cohort of 21,440 individuals participating in the National Physical Fitness Health Test in western China, using a simple random sampling method. To ensure the scientific rigor and validity of the study, participants underwent a thorough screening process. To ensure diagnostic consistency and reliability, all clinical assessors underwent a standardized training program prior to data collection. This training included a comprehensive review of the Fukuda criteria, workshops using case studies to calibrate the interpretation of symptoms, and practice in conducting structured interviews.

Initially, 278 individuals who did not complete the questionnaire and 104 individuals who failed to finish the physical fitness tests were excluded. The final selection was further refined using the following inclusion and exclusion criteria: inclusion criteria: (1) Adults aged 20–59 years; (2) No significant physical disabilities; (3) No chronic or other conditions that could impair physical performance. Exclusion criteria: (1) Pregnant women, to eliminate the potential influence of pregnancy on physical fitness and CFS diagnosis; (2) Individuals with exceptionally high fitness levels, such as fitness enthusiasts, to avoid potential bias; this classification was defined as engaging in structured, high-intensity exercise for more than 5 h per week or regular participation in competitive sports. (3) Individuals with a history of psychological disorders or those currently receiving treatment, to control for confounding effects on CFS symptoms. After applying these criteria, a total of 3,025 participants (1,410 men and 1,615 women) were included in the study. Informed consent was obtained from all participants prior to their involvement, ensuring that they fully understood the study's objectives, methodologies, and any potential risks and benefits. This study was carried out following the principles of the Declaration of Helsinki. The research protocol was reviewed by the Ethics Committee of Ningxia Normal University, and all participants provided written informed consent prior to their participation.

### 2.2 Assessment and classification of chronic fatigue

The diagnosis of CFS in this study was conducted according to the criteria established by the U.S. Centers for Disease Control and Prevention (17). The evaluation process included a comprehensive assessment of physical symptoms, cognitive function, sleep quality, and overall quality of life, all carried out by clinically trained physicians and healthcare professionals to ensure accuracy.

Initially, a thorough medical history and symptom review were conducted for all participants to exclude other potential medical or psychiatric causes of fatigue. Individuals were diagnosed with CFS if they experienced severe, chronic fatigue lasting for at least 6 months, which could not be attributed to any known medical or psychiatric condition, and also exhibited at least four of the following symptoms: significant impairment in short-term memory or concentration that severely affected their ability to work, study, engage in social activities, or maintain personal life quality; recurrent sore throat; tender lymph nodes in the neck or armpits without significant lymphadenopathy; unexplained muscle pain not associated with injury or overexertion; multi-joint pain without redness or swelling; new, persistent, or changed headaches; non-restorative sleep, leaving the individual feeling tired even after adequate rest; and post-exertional malaise lasting more than 24 h following physical activity. These symptoms had to persist or recur during the illness and significantly impair the individual's daily functioning. Patients meeting these criteria were diagnosed with CFS according to the Fukuda standard (17). Occupational category was self-reported and classified into three groups based on the primary nature of work tasks: urban non-manual (primarily desk-based or cognitive work), urban manual (primarily physically demanding labor), and agricultural (farming-related activities). Participants were asked to indicate their main occupation during the past 12 months, and responses were categorized according to pre-defined criteria aligned with national occupational classification standards.

### 2.3 Anthropometric assessment and body composition indicators

Anthropometric measurements were performed by trained examiners following standardized protocols. Body height was measured to the nearest 0.1 cm and body weight to the nearest 0.1 kg, with participants wearing light clothing and no shoes. Body mass index (BMI) was calculated as body weight divided by height squared (kg/m^2^) and used as an indicator of general adiposity. Waist circumference and hip circumference were measured at standard anatomical landmarks, and waist-to-hip ratio (WHR) was computed as an index of central adiposity. These anthropometric indicators were included in the analyses as potential confounders, given their known associations with fatigue-related symptoms and physical performance.

### 2.4 Physical fitness battery and relative performance indicators

Physical fitness was assessed using a multi-domain standardized test battery, designed to capture distinct components of physical capacity relevant to occupational performance and health. The battery included assessments of: cardiorespiratory function: vital capacity (ml) measured via spirometry; Muscle strength: handgrip strength (kg) and back strength (kg); Explosive power: vertical jump height (cm); Muscular endurance: push-ups or knee push-ups (repetitions) and sit-ups (repetitions per minute); Flexibility: sit-and-reach distance (cm); Postural control and balance: one-leg standing with eyes closed (seconds). To minimize confounding by body size and to enhance comparability across individuals, relative performance indicators were additionally derived, including relative handgrip strength (handgrip strength/body weight), relative back strength (back strength/body weight), and relative vital capacity (vital capacity/body weight). These indicators were analyzed descriptively and served as complementary measures of functional capacity.

### 2.5 Construction of composite physical fitness metrics

To capture overall physical fitness while reducing reliance on single test outcomes, a composite Physical Fitness Index was constructed using a Z-score–based approach. Each of the eight physical fitness variables was standardized according to the sample mean and standard deviation. The index was calculated as the arithmetic mean of the standardized scores, with higher values representing better overall fitness. This approach assumes equal contribution of each fitness domain and provides an interpretable global indicator suitable for regression modeling.

### 2.6 Assessment of choice reaction time

Choice reaction time (CRT) was assessed using a computerized two-alternative forced-choice paradigm designed to measure information processing speed and motor response efficiency. The task was programmed using E-Prime 3.0 (Psychology Software Tools, Pittsburgh, PA, USA) and administered on a standardized desktop computer equipped with an Intel i5 processor and a 60 Hz LCD monitor in a quiet testing environment. Participants were seated approximately 60 cm from the screen and were instructed to respond as quickly and accurately as possible to visual stimuli requiring stimulus discrimination. Each trial began with a fixation cross presented for 500 ms, followed by a visual stimulus (a colored circle appearing randomly on either the left or right side of the screen). Participants responded by pressing the corresponding key (“F” for left and “J” for right) on a standard keyboard. The stimulus remained visible until a response was made or until 1,500 ms had elapsed. Inter-trial intervals varied randomly between 800 and 1,200 ms to reduce anticipatory responses. The task included 20 practice trials to ensure comprehension of instructions, followed by 120 formal trials divided into three blocks. Reaction time was recorded with millisecond precision from stimulus onset to keypress. Only correct responses were included in the analysis, and trials with reaction times shorter than 200 ms or longer than 1,500 ms were excluded as anticipatory or delayed responses. The mean reaction time (in milliseconds) across valid correct trials was calculated for each participant and used for statistical analysis. Choice reaction time was conceptualized as a cognitive functional outcome reflecting processing speed and motor execution efficiency rather than as a component of physical fitness.

### 2.7 Statistical analysis

All statistical analyses followed a hierarchical and theory-driven modeling strategy, designed to distinguish between descriptive differences, independent associations, and potential mechanistic pathways. Continuous variables are reported as mean ± standard deviation (SD), and categorical variables as frequencies and percentages. Between-group differences by chronic fatigue status were evaluated using independent-samples *t*-tests for continuous variables and χ^2^ tests for categorical variables. Standardized effect sizes were calculated using Cohen's *d* to facilitate interpretation of group differences.

To identify factors independently associated with chronic fatigue, a series of hierarchical logistic regression models was constructed with chronic fatigue status as the dependent variable. Model 1 included demographic and occupational variables (age, sex, occupational group). Model 2 additionally adjusted for anthropometric indicators (BMI and WHR). Model 3 further incorporated composite physical fitness measures (Physical Fitness Index or PCA-derived components). Results are reported as odds ratios (ORs) with 95% confidence intervals (CIs). Model performance was evaluated using the area under the receiver operating characteristic curve (AUC), and model parsimony was assessed using the Akaike information criterion (AIC).

Associations between chronic fatigue and choice reaction time were examined using multivariable linear regression models. Covariates included age, sex, occupational group, BMI, WHR, and physical fitness measures. Given the large sample size and potential heteroscedasticity, HC3 robust standard errors were applied. Prior to model estimation, multicollinearity among independent variables was assessed using variance inflation factors (VIFs) and tolerance statistics to ensure model stability and independence of predictors. All VIF values were below the commonly accepted threshold of 5, indicating no evidence of problematic multicollinearity. Regression coefficients (β), standard errors, 95% CIs, and *p*-values are reported.

All models were adjusted for demographic, occupational, and anthropometric covariates. Standardized path coefficients were estimated, and indirect effects were calculated as the product of the corresponding paths. Robustness of the main findings was evaluated through multiple sensitivity analyses, including (i) replacement of the Z-score–based Physical Fitness Index with PCA-derived component scores, and (ii) comparison of effect estimates across alternative model specifications. Consistency of results across analyses was interpreted as evidence of robustness. All statistical tests were two-sided. A *p*-value of < 0.05 was considered statistically significant.

## 3 Results

### 3.1 Participant characteristics and prevalence of chronic fatigue

The demographic and clinical characteristics of the participants are summarized in Table 1. A total of 3,025 participants were included in the final analysis. The mean age was 40.03 ± 11.10 years, with 1,698 females (56.1%) and 1,327 males (43.9%). According to occupational classification, 1,423 (47.0%) participants were engaged in urban non-manual work, 1,038 (34.3%) in urban manual work, and 564 (18.6%) in agricultural work.

**Table 1:** Participant characteristics by chronic fatigue status.

**Variable**	**Non-fatigue (*n* = 2,860)**	**Chronic fatigue (*n* = 165)**	***p*-value**
Age, years	40.19 ± 11.12	37.37 ± 10.50	< 0.001
Female, *n* (%)	1,530 (53.5)	93 (56.4)	0.430
Male, *n* (%)	1,330 (46.5)	72 (43.6)	
Urban non-manual, *n* (%)	1,313 (45.9)	110 (66.7)	< 0.001
Urban manual, *n* (%)	993 (34.7)	45 (27.3)	
Agricultural, *n* (%)	554 (19.4)	10 (6.1)	
BMI, kg/m^2^	23.90 ± 2.74	24.22 ± 2.80	0.157
WHR	0.870 ± 0.070	0.879 ± 0.075	0.132

Values are mean ± SD or n (%).

BMI, body mass index; WHR, waist-to-hip ratio.

Based on the pre-defined questionnaire cutoff, 165 participants (5.45%) were classified as having chronic fatigue. The prevalence of chronic fatigue did not differ significantly between females (5.77%) and males (5.05%) (χ^2^ = 0.620, *p* = 0.430). However, chronic fatigue prevalence differed significantly across occupational groups (χ^2^ = 31.633, *p* < 0.001), with the highest prevalence observed in urban non-manual workers (7.73%), followed by urban manual workers (4.34%), and agricultural workers (1.77%).

### 3.2 Differences in physical fitness and reaction time between groups

Descriptive comparisons between participants with and without chronic fatigue are presented in Table 2. Choice reaction time was analyzed as a functional cognitive outcome, whereas physical fitness indicators were treated as measures of physiological capacity. Participants classified as having chronic fatigue exhibited a markedly slower choice reaction time than those without chronic fatigue (641.09 ± 89.70 ms vs. 567.40 ± 75.90 ms). This between-group difference was statistically significant (*t* = 10.34, *p* < 0.001) and corresponded to a large standardized effect size (Cohen's *d* = 0.96), indicating a substantial impairment in functional reaction performance associated with chronic fatigue status.

**Table 2:** Physical fitness and reaction time by chronic fatigue.

**Variable**	**Non-fatigue**	**Chronic fatigue**	***p*-value**	***p*-FDR**	**Cohen's *d***
Choice reaction time, ms	567.40 ± 75.90	641.09 ± 89.70	< 0.001	< 0.001	+0.96
Physical Fitness Index (SD)	0.02 ± 1.01	−0.42 ± 0.75	< 0.001	< 0.001	−0.45
BMI (kg/m^2^)	23.90 ± 2.74	24.22 ± 2.80	0.157	0.236	−0.12
WHR	0.870 ± 0.070	0.879 ± 0.075	0.132	0.226	−0.13
Vital capacity (ml)	3,432.6 ± 1,098.4	3,311.8 ± 1,032.7	0.086	0.172	−0.11
Handgrip strength (kg)	35.99 ± 11.24	33.38 ± 10.76	< 0.001	< 0.001	−0.25
Back strength (kg)	89.23 ± 33.78	80.07 ± 31.46	< 0.001	< 0.001	−0.28
Vertical jump (cm)	26.64 ± 10.43	23.74 ± 9.95	< 0.001	< 0.001	−0.37
Push-ups/knee push-ups (reps)	22.61 ± 13.84	21.04 ± 12.92	0.102	0.198	−0.12
Sit-ups (reps/min)	20.85 ± 10.19	19.43 ± 9.66	0.001	0.003	−0.23
Sit-and-reach (cm)	9.31 ± 7.46	8.62 ± 7.02	0.276	0.345	−0.10
One-leg standing, eyes closed (s)	29.74 ± 21.12	23.32 ± 19.33	< 0.001	< 0.001	−0.37
Waist circumference (cm)	83.61 ± 10.02	84.26 ± 10.90	0.460	0.460	−0.06
Relative handgrip (kg/kg)	0.560 ± 0.135	0.521 ± 0.123	< 0.001	< 0.001	−0.29
Relative back strength (kg/kg)	1.380 ± 0.434	1.235 ± 0.402	< 0.001	< 0.001	−0.34
Relative vital capacity (ml/kg)	43.75 ± 10.33	42.23 ± 9.34	0.045	0.072	−0.15

Values are mean ± SD. Cohen's d was calculated as mean (chronic fatigue)–mean (non-fatigue).

p-values for individual fitness indicators were adjusted using the Benjamini–Hochberg false discovery rate (FDR) method.

To summarize global physical capacity and reduce reliance on single fitness indicators, a Physical Fitness Index was constructed by standardizing eight fitness measures (vital capacity, handgrip strength, back strength, vertical jump, push-ups/knee push-ups, sit-ups, sit-and-reach, and one-leg standing with eyes closed) into Z-scores and averaging these standardized values, with higher scores indicating better overall fitness. Using this composite metric, participants with chronic fatigue demonstrated significantly poorer overall physical fitness compared with non-fatigued participants (−0.42 ± 0.75 SD vs. 0.02 ± 1.01 SD; *t* = 7.20, *p* < 0.001), corresponding to a moderate effect size (Cohen's *d* = −0.45).

Consistent with the composite index, several individual fitness tests differed significantly between groups, with participants with chronic fatigue generally demonstrating lower performance (Table 2). The most prominent deficits were observed in balance-related performance and neuromuscular power. Specifically, one-leg standing with eyes closed was shorter in the chronic fatigue group (23.32 ± 19.33 s vs. 29.74 ± 21.12 s; *p* < 0.001, *d* = −0.37), and vertical jump height was reduced (23.74 ± 9.95 cm vs. 26.64 ± 10.43 cm; *p* < 0.001, *d* = −0.37). Measures of muscular strength were also significantly lower, including back strength (80.07 ± 31.46 kg vs. 89.23 ± 33.78 kg; *p* < 0.001, *d* = −0.28) and handgrip strength (33.38 ± 10.76 kg vs. 35.99 ± 11.24 kg; *p* < 0.001, *d* = −0.25). Muscular endurance, assessed by sit-up performance, showed a modest but significant reduction (19.43 ± 9.66 vs. 20.85 ± 10.19 reps/min; *p* = 0.001, *d* = −0.23).

In contrast, body composition indicators showed only small and non-significant differences between groups. Neither BMI (24.22 ± 2.80 vs. 23.90 ± 2.74 kg/m^2^; *p* = 0.157) nor waist-to-hip ratio (0.879 ± 0.075 vs. 0.870 ± 0.070; *p* = 0.132) differed significantly between participants with and without chronic fatigue. Overall, these findings indicate that chronic fatigue status is associated with both clinically meaningful slowing of reaction performance and broad, multi-domain reductions in physical fitness, particularly in balance, explosive power, and strength-related domains, while differences in general body composition are comparatively small at the descriptive level. Results for individual fitness components remained robust after correction for multiple comparisons using the false discovery rate approach.

### 3.3 Multivariable logistic regression for chronic fatigue

Multivariable logistic regression models were constructed with chronic fatigue as the dependent variable (Table 3). Model performance improved progressively with the inclusion of body composition and physical fitness variables. The area under the ROC curve (AUC) increased from 0.629 (Model 1) to 0.725 (Model 3), accompanied by a substantial reduction in Akaike Information Criterion (AIC). In the fully adjusted model (Model 3), a higher Physical Fitness Index was strongly and independently associated with a lower likelihood of chronic fatigue (OR = 0.303 per SD increase; 95% CI: 0.222–0.412; *p* = 3.34 × 10^−14^). Compared with urban non-manual workers, agricultural workers (OR = 0.177; 95% CI: 0.091–0.343; *p* = 2.97 × 10^−7^) and urban manual workers (OR = 0.520; 95% CI: 0.362–0.748; *p* = 4.14 × 10^−4^) exhibited significantly lower odds of chronic fatigue. Increasing age was inversely associated with chronic fatigue risk (OR = 0.969 per year; 95% CI: 0.952–0.986; *p* = 3.50 × 10^−4^). Male sex was associated with a higher risk of chronic fatigue (OR = 3.443; 95% CI: 2.022–5.863; *p* = 5.30 × 10^−6^). BMI and WHR were not independently associated with chronic fatigue after accounting for physical fitness.

**Table 3:** Multivariable logistic regression models.

**Variable**	**Model 1 OR (95% CI)**	**Model 2 OR (95% CI)**	**Model 3 OR (95% CI)**
Age (per year)	0.973 (0.958–0.988)^***^	0.971 (0.955–0.987)^***^	0.969 (0.952–0.986)^***^
Male sex	1.12 (0.83–1.51)	1.48 (1.03–2.14)^*^	3.44 (2.02–5.86)^***^
Urban manual	0.61 (0.44–0.85)^***^	0.57 (0.40–0.80)^**^	0.52 (0.36–0.75)^***^
Agricultural	0.24 (0.13–0.43)^***^	0.22 (0.12–0.41)^***^	0.18 (0.09–0.34)^***^
BMI (kg/m^2^)		1.03 (0.98–1.08)	1.02 (0.96–1.09)
WHR (per 0.1)		1.21 (0.92–1.60)	1.17 (0.88–1.55)
Physical Fitness Index (per SD)			0.30 (0.22–0.41)^***^
AUC	0.629	0.647	0.725
AIC	1,254.33	1,251.01	1,187.85

^*^p < 0.05; ^**^p < 0.01; ^**^p < 0.001.

Reference group for occupation: urban non-manual.

### 3.4 Association between chronic fatigue and choice reaction time

The multivariable regression results for choice reaction time are summarized in Table 4. In linear regression models adjusted for age, sex, occupational group, BMI, and WHR, chronic fatigue was associated with a significant prolongation of choice reaction time (β = +74.19 ms, *p* < 0.001). After further adjustment for the Physical Fitness Index, chronic fatigue remained independently associated with slower reaction time (β = +69.75 ms; SE = 6.42; 95% CI: 57.17–82.32; *p* = 1.62 × 10^−27^). Higher physical fitness was independently associated with faster reaction time (β = −11.66 ms per SD increase; 95% CI: −15.43 to −7.88; *p* = 1.40 × 10^−9^). The attenuation of the association between physical fitness and reaction time after inclusion of chronic fatigue indicates that chronic fatigue may statistically account for part of the association between physical fitness and cognitive reaction performance. However, given the cross-sectional design, these findings should be interpreted as associative rather than causal.

**Table 4:** Linear regression models for choice reaction time (ms).

**Variable**	**β (SE)**	**95% CI**	***p*-value**
Chronic fatigue (yes vs. no)	+69.75 (6.42)	57.17 to 82.32	1.62 × 10^−27^
Physical Fitness Index (per SD)	−11.66 (1.92)	−15.43 to −7.88	1.40 × 10^−9^
Age (per year)	+1.21 (0.09)	1.04 to 1.39	< 0.001
Male sex	−18.34 (1.87)	−22.01 to −14.67	< 0.001
Urban manual	+6.12 (2.41)	1.39 to 10.85	0.011
Agricultural	+9.48 (3.06)	3.48 to 15.49	0.002
BMI (kg/m^2^)	+0.63 (0.31)	0.03 to 1.23	0.039
WHR (per 0.1)	+4.72 (3.89)	−2.91 to 12.35	0.225

Receiver operating characteristic (ROC) analyses were conducted to evaluate the discriminatory ability of selected health-related physical fitness indicators for distinguishing individuals with and without chronic fatigue. Analyses were performed separately for males and females to account for sex-specific differences in physical capacity and performance. For indicators reflecting physical performance (grip strength, back strength, vertical jump, push-ups, and single-leg stance with eyes closed), lower values were considered indicative of higher chronic fatigue risk, whereas higher values of waist-to-hip ratio (WHR) and choice reaction time (CRT) were considered indicative of higher risk.

**Figure 1:**
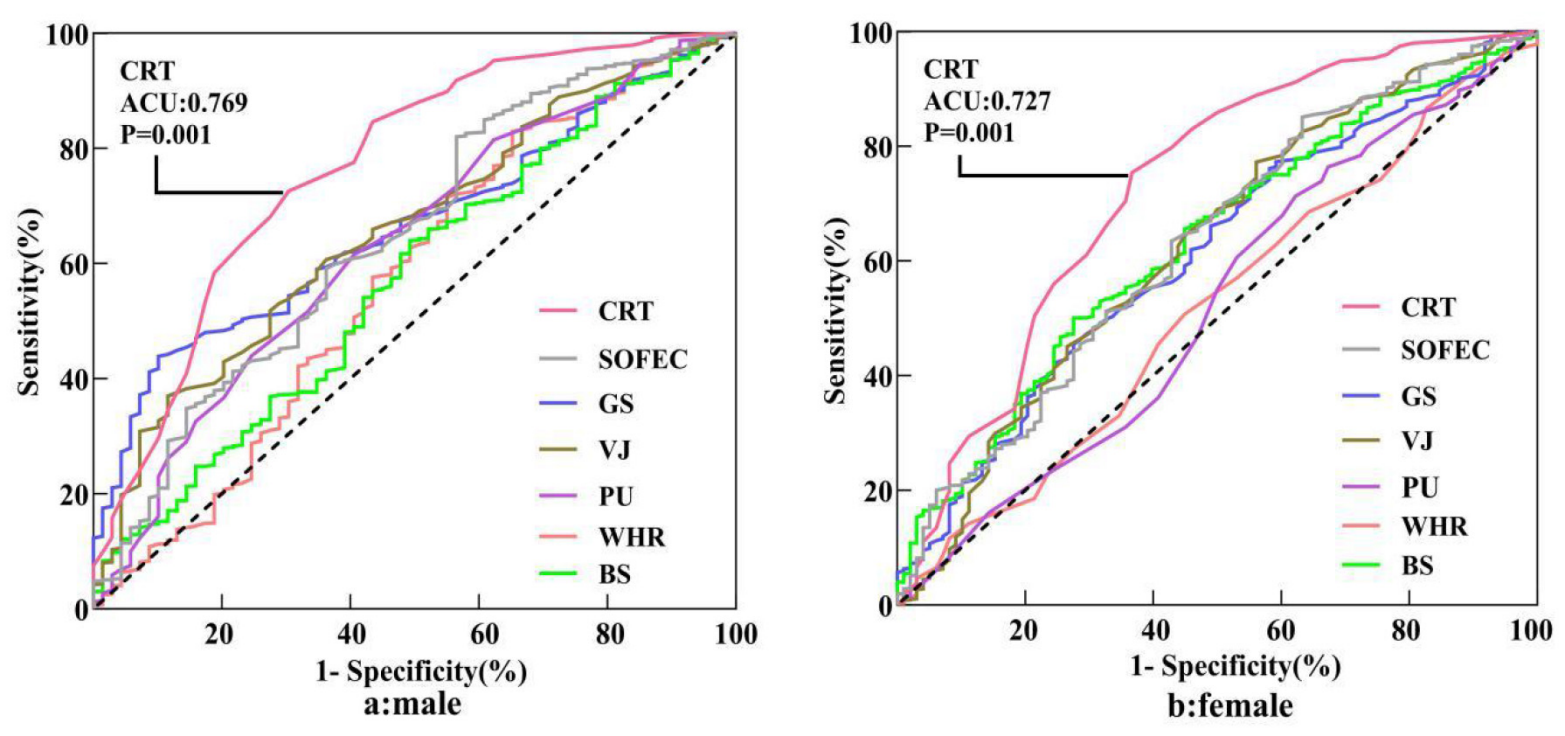
Receiver operating characteristic (ROC) curves depicting the ability of selected health-related physical fitness indicators to distinguish CFS status in this cross-sectional sample.

The sex-specific optimal cutoff values, sensitivities, and specificities for each physical fitness indicator are detailed in Table 5. In the male cohort, several physical fitness indicators demonstrated statistically significant discriminatory ability (Figure 1). The area under the ROC curve (AUC) ranged from 0.574 to 0.761. Among all examined indicators, choice reaction time exhibited the strongest discriminatory performance (AUC = 0.761), followed by vertical jump (AUC = 0.653), single-leg stance with eyes closed (AUC = 0.645), push-ups (AUC = 0.643), and grip strength (AUC = 0.626). Back strength showed relatively weak discriminatory ability (AUC = 0.574). Using the Youden index, the optimal cutoff value for choice reaction time in males was 0.590 s, corresponding to a sensitivity of 0.687 and a specificity of 0.725 (Youden index = 0.411). The optimal cutoff for single-leg stance with eyes closed was 19.50 s (sensitivity = 0.433, specificity = 0.826), and for grip strength was 46.40 kg (sensitivity = 0.791, specificity = 0.442).

**Table 5:** Optimal threshold values of selected health-related physical fitness indicators for distinguishing individuals with and without chronic fatigue.

**Gender**	**Indicators**	**AUC**	**Cut-off**	**Sensitivity**	**Specificity**	**Jorden index**
Male	WHR	0.601	0.889	0.761	0.428	0.189
	GS (kg)	0.626	46.40	0.791	0.442	0.233
	BS (kg)	0.574	111.00	0.507	0.640	0.147
	VJ (cm)	0.653	33.60	0.881	0.369	0.250
	PU (reps)	0.643	19.00	0.612	0.616	0.228
	SOFEC (s)	0.645	19.50	0.433	0.826	0.259
	CRT (s)	0.761	0.590	0.687	0.725	0.411
Female	WHR	0.516	0.853	0.541	0.556	0.097
	GS (kg)	0.597	28.40	0.724	0.440	0.164
	BS (kg)	0.630	65.20	0.724	0.501	0.225
	VJ (cm)	0.626	18.60	0.439	0.773	0.211
	PU (reps)	0.481	18.00	0.357	0.690	0.047
	SOFEC (s)	0.630	19.30	0.367	0.851	0.218
	CRT (s)	0.727	0.640	0.633	0.754	0.387

WC, waist circumference; HC, hip circumference; WHR, waist-to-hip ratio; VC, vital capacity; GS, grip strength; BS, back strength; VJ, vertical jump height; PU, push-up count; SU, sit-up count; SAR, sit-and-reach distance; SOFEC, single-leg stance time with eyes closed; CRT, choice reaction time.

WHR and CRT: higher values indicate higher chronic fatigue risk (positive if ≥ cut-off).

GS/BS/VJ/PU/SOFEC: lower values indicate higher chronic fatigue risk (positive if ≤ cut-off).

SOFEC unit is seconds (s).

CRT unit is seconds (s).

In the female cohort, the discriminatory performance of physical fitness indicators was generally modest but statistically significant for several measures. The highest AUC was again observed for choice reaction time (AUC = 0.727), followed by back strength (AUC = 0.630), single-leg stance with eyes closed (AUC = 0.630), and vertical jump (AUC = 0.626). Grip strength demonstrated moderate discriminatory ability (AUC = 0.597), whereas waist-to-hip ratio and push-ups showed limited or no discriminatory value (AUC = 0.516 and 0.481, respectively). The optimal cutoff for choice reaction time in females was 0.640 s, yielding a sensitivity of 0.633 and a specificity of 0.754 (Youden index = 0.387). The optimal cutoff for single-leg stance with eyes closed was 19.30 s (sensitivity = 0.367, specificity = 0.851).

Overall, ROC analyses indicated that choice reaction time consistently outperformed individual physical fitness indicators in discriminating chronic fatigue status in both sexes. Balance-related performance and explosive power measures demonstrated moderate discriminatory ability, whereas anthropometric indicators such as WHR showed limited predictive value. These findings suggest that functional cognitive performance, as indexed by reaction time, may serve as a sensitive marker of chronic fatigue, complementing traditional physical fitness assessments.

## 4 Discussion

This study provides a large-scale, multidimensional evaluation of the relationship between health-related physical fitness and CFS in a community-based adult population. The principal finding is that impairments in muscular strength, neuromuscular control, and cognitive–motor processing speed are consistently and independently associated with CFS status across sexes. In particular, choice reaction time demonstrated the strongest discriminatory performance, suggesting that central processing speed may represent a sensitive functional marker of fatigue-related impairment. These findings extend existing literature by integrating composite fitness profiling with functional cognitive outcomes within a single analytic framework, highlighting that chronic fatigue is characterized not only by subjective symptoms but also by measurable reductions in neuromuscular and central performance capacity.

The most significant contribution of this study lies in establishing a series of quantifiable objective phenotypes for CFS through standardized physical fitness assessments. Compared to healthy controls, individuals with CFS demonstrated substantial impairments across multiple physiological domains, including grip strength, vertical jump, single-leg stance with eyes closed, and choice reaction time. This multi-system impairment pattern provides compelling evidence for CFS as a complex, biologically-grounded disorder rather than a purely psychological condition (18). The observed decline in grip strength represents more than simple muscular weakness. Recent investigations have revealed significant mitochondrial abnormalities in CFS patients, including impaired oxidative phosphorylation capacity and reduced ATP production (19). These cellular-level energy deficits likely explain the disproportionate fatigability and force reduction observed during sustained muscle contraction (20). The consistency between our objective grip strength measurements and previous reports of metabolic dysfunction strengthens the biological validity of CFS as a distinct medical entity. Perhaps the most striking finding concerns choice reaction time, which demonstrated the strongest discriminatory performance for differentiating CFS status in both genders. Choice reaction time reflects the integrity of complex neural processes spanning sensory integration, cognitive processing, and motor execution, thereby providing an objective index of central nervous system function (21). Neuroimaging studies have consistently identified functional and structural abnormalities in CFS patients within brain networks critical for information processing, including the pre-frontal cortex, anterior cingulate, and basal ganglia (22). The pronounced prolongation of reaction times in our CFS cohort likely reflects these underlying neural disruptions, providing an objective correlate for the subjective experience of brain fog that has been notoriously difficult to quantify (23).

Equally informative are the physical fitness parameters that showed no significant differences between CFS patients and healthy controls. The absence of group differences in BMI, hip circumference, vital capacity, and sit-and-reach performance provides crucial insights for differential diagnosis and pathophysiological understanding. The preserved vital capacity despite marked exercise intolerance suggests that CFS-related fatigue does not primarily stem from cardiopulmonary limitations. This finding aligns with the effort perception model, wherein CFS patients demonstrate normal cardiopulmonary function but experience disproportionately high perceived exertion during physical tasks. This mismatch between objective capacity and subjective experience may reflect central nervous system dysregulation rather than peripheral organ dysfunction (24). Furthermore, the normal performance on the sit-and-reach test indicates preserved connective tissue elasticity, helping distinguish CFS from conditions like fibromyalgia where widespread pain and tissue sensitivity are pre-dominant features (25). These differential impairment patterns enhance our ability to discriminate CFS from other fatigue-related conditions in clinical practice.

Our findings reveal sex-specific patterns in the association between physical fitness parameters and CFS risk. These differences should be interpreted cautiously as empirically observed patterns within the present sample rather than definitive biological distinctions. The observed sexual dimorphism may reflect the interaction between physiological reserve capacity and gendered occupational and activity exposures, consistent with socio-ecological and role-based models of health behavior. According to the reserve capacity framework, vulnerability arises when environmental demands exceed domain-specific physiological capacity. Thus, these findings should be regarded as hypothesis-generating and warrant confirmation in longitudinal and mechanistic studies incorporating detailed workload and activity assessments. In Western China, traditional gender roles continue to shape occupational exposures and physical activity patterns. Men often engage in occupations requiring sustained cardiorespiratory endurance and core strength, making these physiological capacities particularly relevant to their functional reserve (26). Conversely, women frequently perform domestic and agricultural tasks demanding repetitive lifting, bending, and squatting, placing greater emphasis on lower back strength and lower limb power (27). This pattern aligns with the reserve capacity model, which posits that disease vulnerability emerges when environmental demands exceed an individual's physiological capacity in specific domains (28). Our findings suggest that CFS risk may be highest when individuals face demands that challenge their most vulnerable physiological systems, with these vulnerabilities being shaped by both biological pre-dispositions and socially-constructed activity patterns.

The occupational gradient observed in our study, with CFS prevalence increasing from farmers (2.1%) to urban manual workers (4.6%) to urban non-manual workers (7.5%), challenges simplistic associations between physical exertion and disease risk. While farmers experience high physical activity levels, their work typically occurs in structured, autonomous contexts with natural pacing. In contrast, urban non-manual workers face the modern health challenge of combining sedentary behavior with high cognitive demands and low job control. Low job control refers to limited autonomy in decision-making, reduced flexibility in task pacing, and restricted authority over work scheduling, as conceptualized in the Job Demand–Control model (29). Cognitive–motor slowing refers to delayed integration of sensory processing, central decision-making, and motor execution. Neurobiologically, such slowing has been linked to alterations in cortical excitability, reduced pre-frontal efficiency, and dysregulation of central fatigue pathways, including dopaminergic and inflammatory signaling mechanisms. Prior neurocognitive studies in fatigue-related conditions have consistently reported slowed processing speed as a core functional impairment. This occupational pattern strongly implicates chronic psychosocial stress as a potential trigger for CFS, possibly through dysregulation of the hypothalamic-pituitary-adrenal axis and promotion of systemic inflammation (30, 31). The higher prevalence in urban non-manual workers may be related to sustained cognitive demands and prolonged sedentary work patterns inherent in many white-collar occupations (32–34). The robust associations between physical fitness indicators and CFS status hold significant promise for clinical practice. However, these interpretations remain inferential, as detailed measures of occupational workload, psychosocial stress, and neuroendocrine function were not directly assessed in the present study and should be formally tested in future longitudinal or mixed-methods research.

Although several health-related physical fitness indicators demonstrated statistically significant associations with CFS status, their practical relevance should be interpreted primarily in terms of effect magnitude and discriminatory performance rather than statistical significance alone. The observed odds ratios for grip strength, single-leg stance with eyes closed, and choice reaction time indicate modest-to-moderate associations, suggesting that impairments in these domains are meaningfully related to CFS status but are insufficient to fully account for disease occurrence on their own. From a discrimination perspective, the area under the AUC values for most indicators ranged from approximately 0.60 to 0.77, reflecting fai, but not excellent, ability to distinguish individuals with and without CFS. Notably, choice reaction time consistently demonstrated the highest AUC across genders, whereas traditional strength measures such as back strength showed comparatively weaker discriminatory performance. This pattern suggests that indicators capturing central processing speed may be more sensitive to CFS-related dysfunction than general musculoskeletal measures.

Importantly, the combination of relatively high sensitivity and modest specificity observed for several indicators has direct implications for practical application. In public health and community-based contexts, higher sensitivity is advantageous for initial screening, as it reduces the likelihood of missing individuals who may have CFS. Conversely, the modest specificity indicates that these measures should not be used as standalone diagnostic tools, but rather as pre-liminary filters to identify individuals who may benefit from further clinical evaluation. Taken together, these findings suggest that simple physical fitness assessments, particularly grip strength and choice reaction time, may have practical value as low-cost, scalable screening aids or as components of multi-domain assessment frameworks, rather than as definitive clinical diagnostic instruments. Their utility is therefore greatest when integrated with symptom-based questionnaires and clinical judgment, especially in population-level or resource-limited screening settings.

Therefore, while choice reaction time is a powerful research tool and a compelling objective marker, its current form may not be directly transferable to widespread clinical practice. Future research may explore the feasibility of technology-assisted reaction time assessments; however, such approaches require rigorous validation against laboratory-grade systems to ensure adequate temporal precision before clinical adoption. Grip strength measurement represents a particularly viable screening tool due to its simplicity, low cost, and high sensitivity. However, the generally modest specificity of all indicators suggests they are best employed as initial screening measures rather than definitive diagnostic tools (35). Future research should prioritize the development of integrated assessment protocols that combine the most promising physical fitness measures with validated symptom inventories. The development of smartphone-based applications for standardized reaction time and balance assessment could dramatically improve accessibility and feasibility in diverse clinical settings (36).

Several limitations warrant consideration when interpreting our findings. The cross-sectional design precludes causal inference regarding the observed associations, whether physical fitness deficits pre-dispose to CFS development, result from the condition, or reflect shared underlying mechanisms remains uncertain. The regional sampling frame, while providing valuable insights into Western China, may limit generalizability to other populations with different different ancestral and biological profiles, cultural contexts, and healthcare systems. Furthermore, we were unable to account for potential confounding by unmeasured variables such as specific comorbidities, medication use, or detailed dietary patterns that might influence both physical fitness and CFS risk.

## 5 Conclusion

This study demonstrates that chronic fatigue is associated with clinically meaningful slowing of cognitive–motor processing and multidimensional impairments in physical fitness among working-age adults. Lower overall physical fitness was independently associated with higher likelihood of chronic fatigue. Although causal direction cannot be determined, these findings suggest that reduced physiological reserve and fatigue-related central slowing may coexist within a shared functional pathway.

## Data Availability

The raw data supporting the conclusions of this article will be made available by the authors, without undue reservation.
